# Heparin binding epidermal growth factor–like growth factor is a prognostic marker correlated with levels of macrophages infiltrated in lung adenocarcinoma

**DOI:** 10.3389/fonc.2022.963896

**Published:** 2022-11-10

**Authors:** Nguyen Van Hiep, Wei-Lun Sun, Po-Hao Feng, Cheng-Wei Lin, Kuan-Yuan Chen, Ching-Shan Luo, Le Ngoc Dung, Hoang Van Quyet, Sheng-Ming Wu, Kang-Yun Lee

**Affiliations:** ^1^ International Ph.D. Program in Medicine, College of Medicine, Taipei Medical University, Taipei, Taiwan; ^2^ Oncology Center, Bai Chay Hospital, Quang Ninh, Ha Long, Vietnam; ^3^ Department of Thoracic and Neurological Surgery, Bai Chay Hospital, Quang Ninh, Ha Long, Vietnam; ^4^ Division of Pulmonary Medicine, Department of Internal Medicine, Shuang Ho Hospital, Taipei Medical University, New Taipei City, Taiwan; ^5^ Division of Pulmonary Medicine, Department of Internal Medicine, School of Medicine, College of Medicine, Taipei Medical University, Taipei, Taiwan; ^6^ TMU Research Center for Thoracic Medicine, Taipei Medical University, Taipei, Taiwan; ^7^ Department of Biochemistry and Molecular Cell Biology, School of Medicine, College of Medicine, Taipei Medical University, Taipei, Taiwan; ^8^ Graduate Institute of Clinical Medicine, College of Medicine, Taipei Medical University, Taipei, Taiwan

**Keywords:** heparin-binding EGF-like growth factor (HBEGF), bioinformatics, biomarker, immune infiltration, macrophage chemotaxis, non-small cell lung cancer

## Abstract

**Background:**

The interactions between tumor cells and the host immune system play a crucial role in lung cancer progression and resistance to treatment. The alterations of EGFR signaling have the potential to produce an ineffective tumor-associated immune microenvironment by upregulating a series of immune suppressors, including inhibitory immune checkpoints, immunosuppressive cells, and cytokines. Elevated Heparin-binding EGF-like growth factor (HB-EGF) expression, one EGFR ligand correlated with higher histology grading, worse patient prognosis, and lower overall survival rate, acts as a chemotactic factor. However, the role of heparin-binding epidermal growth factor-like growth factor (HB-EGF) in the accumulation of immune cells in the tumor microenvironment remains unclear.

**Methods:**

The clinical association of HB-EGF expression in lung cancer was examined using the Gene Expression Omnibus (GEO) repository. HB-EGF expression in different cell types was determined using single-cell RNA sequencing (scRNA-seq) dataset. The correlation between HB-EGF expression and cancer-immune infiltrated cells was investigated by performing TIMER and ClueGo pathways analysis from TCGA database. The chemotaxis of HB-EGF and macrophage infiltration was investigated using migration and immunohistochemical staining.

**Results:**

The high HB-EGF expression was significantly correlated with poor overall survival in patients with lung adenocarcinoma (LUAD) but not lung squamous cell carcinoma (LUSC). Moreover, HB-EGF expression was correlated with the infiltration of monocytes, macrophages, neutrophils, and dendritic cells in LUAD but not in LUSC. Analysis of scRNA-seq data revealed high HB-EGF expression in lung cancer cells and myeloid cells. Results from the pathway analysis and cell-based experiment indicated that elevated HB-EGF expression was associated with the presence of macrophage and lung cancer cell migration. HB-EGF was highly expressed in tumors and correlated with M2 macrophage infiltration in LUAD.

**Conclusions:**

HB-EGF is a potential prognostic marker and therapeutic target for lung cancer progression, particularly in LUAD.

## Introduction

Lung cancer has the highest incidence among various types of cancer and is the leading cause of mortality worldwide. Despite advancements in screening, diagnostic, and therapeutic approaches, the overall survival (OS) of patients with lung cancer remains low (1). Immune checkpoint inhibitors, particularly those blocking cytotoxic T-lymphocyte–associated protein 4 (CTLA4) and programmed death-1 (PD-1)/programmed death-ligand 1 (PD-L1), have exhibited promising efficacy against lung cancer, with long-term survival benefits. However, less than 20% of patients respond to those agents, and acquired resistance is observed in many patients (2). Complex networks among tumor cells and the tumor microenvironment (TME) promote cancer progression and chemoresistance (3). The TME consists of cancer cells, associated fibroblasts, tumor vasculature, lymphoid tissue, adipocytes, cytokines, exosomes, and tumor-infiltrating immune cells (TIILs) such as T cells, B cells, dendritic cells (DC), myeloid-derived suppressor cells (MDSCs), and tumor-associated macrophages (TAMs) (4). Gaining insights into these networks can guide the development of current immunotherapies.

Aberrant signaling of ERBB family members plays an important role in tumorigenesis, particularly the epidermal growth factor (EGF) receptor (EGFR) in lung cancer. EGFR ligands that bind to their receptors mediate downstream signaling pathways, including RAS (rat sarcoma)/RAF (rapidly accelerating fibrosarcoma), phosphatidylinositol 3-kinase (PI3K)/AKT (the serine/threonine protein kinase) pathway contributes to lung tumor growth and metastasis (5). Activation of downstream signaling pathways of EGFR may also play an important role in evading antitumor immune responses (6). EGFR ligands, such as EGF and transforming growth factor [TGF]α, reduce tumor antigen presentation through MHC class I and II expression, while EGF promotes M2 polarization of macrophages (7, 8). Approximately 10-30% of patients with NSCLC harbor EGFR mutations, mainly in exons 18-21, which are considered to be oncogenic drivers and highly promote EGFR downstream signaling pathways (9). In addition, EGFR-mutated cancer cells can cause an anti-immune response in the TME by inducing a series of immunosuppressive events, including recruitment of immunosuppressive cells such as macrophages and Tregs, overexpression of suppressive immune checkpoints such as PD-1 and CTLA-4, induction of cytokines and TGF-β, and reduction of anti-tumor immune cells such as cytotoxic T lymphocytes (10, 11). In addition to its role in cancer cells, EGFR is expressed on several immune cells, including macrophages (12, 13), monocytes (14), plasma cells (15), and Tregs (16). Notably, macrophages constitute the bulk of the cells in the immune infiltrate of tumors, and their impact on cancer progression is extremely variable depending on their phenotype in the TME (17). Briefly, M1 and M2 macrophages in the TME can be divided into pro- and anti-inflammatory phenotypes, respectively (18). In lung cancer, M2 macrophage infiltration into tumor islets was associated with poor prognosis of NSCLC patients (19). CD204-positive TAM is the preferred marker for prognostic prediction in LUAD (20). NSCLC patients with EGFR mutations exhibit high triggering receptor expressed on myeloid cells 2 (TREM2)-positive (+) TAM infiltrations with unique NSCLC molecular features and advanced cancer progression (21). Additionally, TREM2+ TAMs are enriched in multiple anti-inflammatory cytokines, exhibit an M2-like immunosuppressive phenotype, and enhance T cell dysfunction. TAM polarization leading to M2 macrophage prevalence in the TME can confer drug resistance (22). M2 type TAM-derived exosomes also contribute to irreversible tyrosine kinase inhibitor (TKI) of EGFR, osimertinib resistance in NSCLC by modulating the MSTRG.292666.16/miR-6386-5p/MAPK8IP3 axis (23). Remarkably, a cannabinoid receptor 2 agonist, JWH-015, inhibits M2 macrophage-induced epithelial-mesenchymal transition (EMT) in NSCLC cells by downregulating the EGFR signaling pathway (24). Furthermore, reprogramming of TAMs from M2 to M1 phenotype overcomes EGFRT790M-mediated drug resistance by dual targeting mannose receptor-overexpressing TAMs and HER-2+ NSCLC cells (25).

Heparin-binding EGF-like growth factor (HB-EGF), which is a high-affinity EGFR ligand, is involved in lung development and plays a vital role in the differentiation of alveolar epithelial type II cells (26). HB-EGF was first found in a conditional medium derived from macrophages. The soluble form of HB-EGF serves as a paracrine and autocrine mitogen factor for fibroblasts (27), smooth muscle cells (28), keratinocytes (29), and some cancer cells such as ovarian (30), cervical (31), and breast cancer cells (32). HB-EGF induces the chemotaxis and recruitment of cells expressing EGFR and human EGF receptor 4, which is associated with PI3K activity (33). HB-EGF cleavage by matrix metalloproteinase 14 may enhance the EGFR signaling pathway to increase cancer cell growth in NSCLC (34) and promotes lung cancer cell proliferation through the signal transducer and activator of transcription 3 (STAT3) signaling pathway (35). High level of HB-EGF in the TME is associated with the activation and accumulation of macrophages, which may promote cancer progression (31). In cervical cancer, HB-EGF is produced primarily in the tumor cell compartment, not in the stroma. Remarkably, TAMs also mediates the expression of HB-EGF and other EGFR ligands to activate EGFR signaling and subsequent tumor cell proliferation (31). CXCL12-driven stimulation of cervical and colon cancer cells and macrophages may initiate and promote the granulocyte-macrophage colony-stimulating factor/HB-EGF paracrine loop, followed by macrophages leading to cancer cell survival (36). Furthermore, elevated HB-EGF expression in lung cancer is correlated with cancer cell growth, higher histology grading, and poor prognosis (35). Apart from tumor growth-promoting effects, the detailed role of HB-EGF in the TME in lung cancer remains largely unknown. Notably, TAM infiltrations and its polarization are associated with lung cancer progression and drug resistance (19–21, 23–25). The autocrine and paracrine actions of HB-EGF derived from TME cancer cells and tumor-infiltrating immune cells (TIICs) may lead to lung cancer progression. Thus, we would further explore whether high HB-EGF expression promotes the tumor progression associated with infiltrating immune cells.

We hypothesized that HB-EGF is part of the immune TME and is associated with poor survival outcomes in patients with lung cancer. We comprehensively analyzed its expression and role in the prognosis of patients with non–small-cell lung cancer (NSCLC), including LUAD and LUSC, in the GEO datasets. Moreover, we evaluated the association of HB-EGF with TIICs in the TCGA NSCLC and Tumor Immune Estimation Resource (TIMER) and verified the role of HB-EGF in TAM migration by using our cohort of patients and *in vitro* studies.

## Materials and methods

### Survival analysis in GEO datasets

Three microarray data sets of NSCLC (GSE30219, GSE3141, and GSE50081) were obtained from the GEO database by using the R package “GEOquery” (37). The differential expression levels of EGFR ligands, namely HB-EGF, EGF, TGF-α, betacellulin [BTC], amphiregulin [AREG], and epiregulin [EREG], were analyzed using the Mann–Whitney–Wilcoxon test. From each database, the data of LUAD and LUSC were selected for survival analysis. The cutoff value for the high and low HB-EGF groups was determined as the median. The survival curves were fitted and visualized using two packages: “survival” and “survminer” in R studio.

### Gene expression and correlation analysis in TCGA database

The mRNA sequencing data of LUAD and LUSC from TCGA were downloaded to investigate the association between the gene of HB-EGF and the gene of immune infiltration. The specific gene markers of each immune cell were referred from the CellMarker database (38). To assess the relationship between HB-EGF and other genes, Spearman’s R correlation coefficient was calculated.

### Analysis of single-cell RNA-sequencing

The single-cell RNA sequencing LUAD data were downloaded from GEO datasets (GSE131907). From expression matrix data, all 15 tumor samples—11 early stages and 4 late stages—were selected (**Supplementary Table 1**; extracted from the original paper) (39). Three quality filter criteria were applied to each cell: mitochondrial gene percentage (≤20%), unique molecular identifiers (100 to 150,000), and gene count (200 to 10,000). The 2000 highest variable genes were identified and used for principal component analysis (PCA)-based dimension reduction. Uniform Manifold Approximation and Projection (UMAP) were used to visualize clusters. Following the original paper, 60,924 cells were clustered into eight major cell lineages by using specific markers: epithelial cells, endothelial cells, fibroblasts, myeloid cells, B lymphocytes, T lymphocytes, NK cells, and mast cells (39) (**Supplementary Table 2**).

### Immune infiltration analysis by TIMER2.0

The correlation between HB-EGF expression and the number of immune infiltrates, including those of T cells, B cells, NK cells, neutrophils, monocytes, macrophages, dendritic cells, myeloid-derived suppressive cells, mast cells, and cancer-associated fibroblasts, were analyzed across the LUAD and LUSC databases. We adjusted for Spearman’s correlations and purity. The algorithms TIMER, EPIC, MCP-COUNTER, CIBERSORT, CIBERSORT-ABS, XCELL, and QUANTISEQ provided by TIMER 2.0 (http://timer.cistrome.org/) were applied to evaluate immune infiltration.

### Pathway analysis

We analyzed 576 samples from LUAD and evaluated the correlation with HB-EGF. The RNA-seq data (Level 3) were normalized with the Log2 (RSEM+1) method. The most correlated genes (n = 182) were used for pathway analysis performed using ClueGo software (**Supplementary Table 3**). The strength of the association between the terms was determined using kappa statistics. The network was generated and visualized using the Cytoscape yFiles radial layout.

### Cell culture and differentiation

The human monocytic THP-1 and U-937 cells (ATCC, Manassas, VA, USA) were suspension cultured in RPMI 1640 medium (Gibco-11875) containing 10% fetal bovine serum and supplemented with 100 U/mL penicillin, 100 μg/mL streptomycin, and 50 μM β-mercaptoethanol. For stimulating the differentiation of THP-1 cells into macrophages, 10 ng/mL phorbol 12-myristate 13-acetate (PMA; Sigma-Aldrich, St. Louis, MO, USA) was added in culture medium for 48 h.

### Migration assay

A monocyte migration assay was performed to investigate the chemotactic function of HB-EGF. First, 200 μL of serum-free RPMI 1640 containing 5 × 10^5^ THP-1 cells were added to the upper chamber (24-transwell inserts, pore size of 8 μm, Corning, NY, USA). Subsequently, 600 μL of serum-free RPMI 1640 medium containing 100 ng/mL HB-EGF (Peprotech, Rocky Hill, NJ, USA) was loaded into the bottom chamber. The migrated cells were observed using an inverted microscope and counted in four fields (200× magnification) at 2, 4, and 8 h. This experiment was repeated three times.

After 10^5^ THP-1 cells were differentiated into macrophages in the upper chamber for 48 h, the medium was discarded, and the cells were washed twice with phosphate-buffered saline (PBS) before using them in the migration assay. The same dose of HB-EGF was used in the bottom chamber to attract macrophages for 8 h. The non-migrated cells were gently scraped and washed with PBS. The migrated cells were fixed in ethanol and stained with crystal violet. At least four pictures of the migrated cells were obtained at 200× magnification and quantified using FIJI software. The experiments were repeated three times.

### Macrophages cocultured with lung cancer cells

The macrophage-mediated cell proliferation of lung cancer was performed using a coculture assay. To stimulate differentiation into macrophages, 2 × 10^5^ U-937 monocytes were seeded into the upper chamber of the transwell (porous with 0.4-μm pores; Corning, NY, USA) and treated with 10 ng/mL PMA for 24 h. Subsequently, cells were cultured in a medium with 20 ng/mL interleukin-4 (IL-4) and IL-13 for an additional 48 h to induce M2 macrophages. Cells were then washed with PBS and incubated with RPMI medium containing 2 μg/ml control IgG or HB-EGF antibodies (AB clonal, A16365) for 24 h. On the same day, 2 × 10^5^ A549 cells were cultured in the lower chamber and incubated for 24 h to allow attachment. The subsequent coculture cells were incubated in a 6-well plate for 24–72 h. To assess M2 macrophage (ϕ)-mediated cancer cell migration, 1 × 10^5^ U-937 monocytes were seeded in the 24-well plate. Similarly, cells were treated with PMA and M2 macrophage differentiation was subsequently applied. Then, M2 macrophages were washed with PBS and incubated with control or HB-EGF antibodies containing medium for 24 h. Subsequently, 1 × 10^5^ A549 cells were seeded in the upper chamber (8-μm pore size) and placed on top of the 24-well plate containing M2 macrophages or a regular RPMI medium-only control. After 24 h of stimulation, migrated cells were stained, counted, and compared with control cells.

### Immunohistochemistry

We obtained 30 tissue specimens of LUAD from the biobank at Taipei Medical University–Shuang Ho Hospital. **Supplementary Table 4** lists clinical information. Immunohistochemical (IHC) staining was performed using 4-μm tissue sections. The tissues were deparaffinized and rehydrated by immersing them in a series of xylene and graded alcohol. Antigen retrieval was applied using Tris-EDTA buffer (pH 9.0) by pressuring cooking the tissue slides for 10 minutes. Endogenous peroxidase activity was blocked with 0.3% H_2_O_2_ for 15 min, and the tissues were incubated for 1 h in 10% bovine serum albumin to prevent nonspecific conjugation. For HB-EGF staining, the slides were incubated for 1 h at room temperature with the HB-EGF rabbit polyclonal antibody (AB clonal, A16365, 1:200 dilution). The slides were washed three times with PBS-T and incubated with the secondary antibody for 20 min. The color was developed using alkaline phosphatase (AP, EnzoBioscience). For the macrophage phenotype, double staining was performed using the mouse anti-CD68 monoclonal antibody (Proteintech, 66231-2-Ig, 1000 µg/mL, 1:1000) and rabbit anti-iNOS polyclonal antibody (Proteintech, 18985-1-AP, 267 µg/mL, 1:500) for M1 macrophage staining and the CD68 antibody (Proteintech, 66231-2-Ig, 1000 µg/mL, 1:1000) and CD163 polyclonal antibody (Proteintech, 16646-1-AP, 500 µg/mL, 1:200) for M2 macrophage staining. The primary antibodies were incubated for 1 h at room temperature, and the color was developed using AP and diaminobenzidine (Enzo, polyview, ADI-950-100) for 20 and 5 min, respectively. The single staining and negative control were performed but not shown in the context. CD68 is a pan-macrophage marker and CD163 is a M2 macrophage marker. M1-like macrophages, characterized by CD68 expression. iNOS is a M1 macrophage marker. The polarization macrophage can be categorized as: CD68^+^/CD163^-^/iNOS^-^ (M0); CD68^+^/CD163^+^ cells (M2) and CD68^+^/iNOS^+^ (M1). The negative control was performed with the secondary antibodies (mouse and rabbit isotope controls). Negative control was always performed along with each experiment. Hematoxylin was applied as a counterstain for 5 min before dehydrating and covering the slides.

### IHC quantification

HB-EGF expression was semi-quantitatively evaluated using the H-score (40), which was calculated by multiplying the percentage of positive cells with different staining intensity values (0: no signal, 1: weak, 2: moderate, and 3: strong). The proportion of positive cells was calculated using FIJI software, with double-stained cells identified using the Trainable Weka Segmentation, an integrated machine learning tool in FIJI (41). The percentage of double-positive cells represented the macrophage fraction in the tumor. At least four fields at 200× magnification from each slide were used for macrophage quantification.

## Results

### Higher HB-EGF gene expression predicted poor prognosis in LUAD

**Figure 1:**
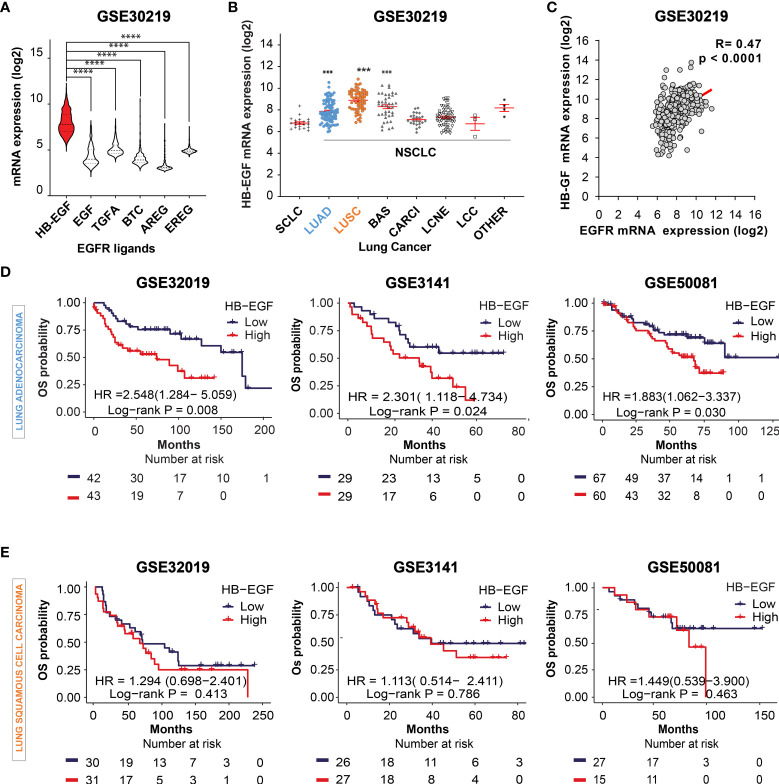
Prognostic potential of HB-EGF expression in LUAD **(A)** The gene expression levels of EGFR ligands, namely heparin-binding EGF-like growth factor (HB-EGF), epidermal growth factor (EGF), transforming growth factor-α (TGF-α), betacellulin (BTC), amphiregulin (AREG), and epiregulin (EREG), in lung cancer were analyzed using the GSE30219 dataset. The levels of HB-EGF were compared with those of other ligands by using the Wilcoxon rank-sum test, ****P < 0.0001. **(B)** Expression of the HB-EGF mRNA level across different types of lung cancer, including lung small cell carcinoma (SCLC), lung adenocarcinoma (LUAD), lung squamous cell carcinoma (LUSC), lung cancer basaloid (BAS), lung cancer carcinoid (CARCI), lung cancer large cell neuroendocrine (LCNE), and large cell carcinoma (LCC) in GSE 30219 ***p < 0.001. **(C)** The correlation between the gene expression of HB-EGF and EGFR in GSE30219 was determined by performing Spearman’s rank correlation analysis. **(D, E)** Kaplan–Meier plot analysis of overall survival (OS) according to the HB-EGF mRNA expression level in patients with LUAD and LUSC in different GEO datasets (GSE30219, GSE3141, and GSE50081). We calculated p values by using the log-rank test.

Activation of EGFR ligands and their downstream signaling pathways play a critical role in lung cancer progression (42). First, we compared the mRNA levels of common EGFR ligands in lung cancer (HB-EGF, EGF, TGF-α, BTC, AREG, and EREG) by using the gene expression dataset GSE30219, GSE3141, and GSE50081. HB-EGF exhibited the highest expression among the examined EGFR ligands (**Figure 1A** and **Supplementary Figure 1A**). Moreover, among the distinct subtypes of lung cancer cells, the HB-EGF mRNA level was highly expressed in NSCLC, especially in LUAD, LUSC, and basaloid squamous cell carcinoma (BAS), compared with small-cell lung cancer (SCLC; **Figure 1B**). In addition, the dot plot revealed a positive correlation between HB-EGF and EGFR gene expression levels in NSCLC (r = 0.470, p < 0.0001; **Figure 1C** and **Supplementary Figures 1B–D**). These results suggest that higher HB-EGF expression may play a vital role in lung cancer progression, particularly in NSLC.

To investigate the prognostic role of HB-EGF, we performed Kaplan–Meier survival analysis for determining the relationship between HB-EGF expression and OS in patients with NSCLC, particularly LUAD and LUSC. Among the patients with LUAD, those with higher HB-EGF expression had poorer OS in several GEO datasets, including GSE30219 (HR and 95% CI = 2.548 [1.284–5.059], p = 0.008), GSE3141 (HR = 2.301 [1.118–4.734], p = 0.024), and GSE32019 (HR = 1.883 [1.062–3.337], p = 0.030) (**Figure 1D**). No significant correlations were observed between HB-EGF expression and OS in patients with LUSC in these datasets (GSE30219: HR = 1.294 [0.698–2.401], p = 0.413; GSE3141: HR = 1.113 [0.514–2.411], p = 0.786; and GSE50081: HR = 1.449 [0.539–3.900], p = 0.463) (**Figure 1E**). Interestingly, by analyzing 4 GEO data including GSE29013, GSE31210, GSE50081, GSE8894, we found that the high HB-EGF expression relate with shorter progression free survival time in both LUAD and LUSC (**Supplementary Figure 2**). Collectively, our results indicated that the patients with higher HB-EGF expression had poorer prognosis, specially in LUAD.

### HB-EGF expression was associated with tumor immune infiltration cell

**Figure 2:**
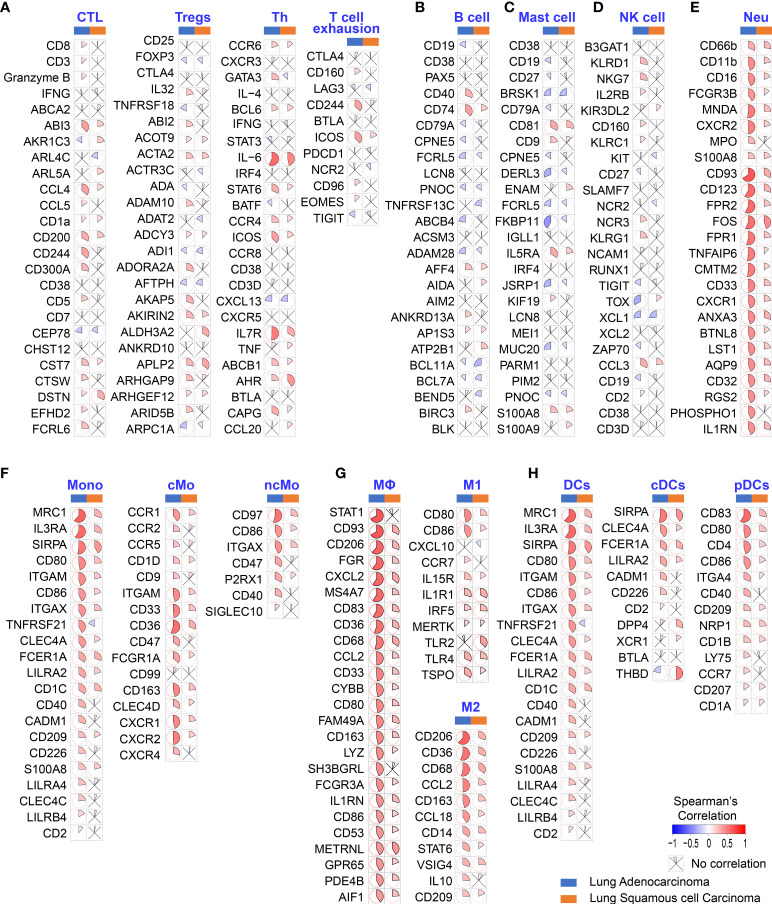
Correlation between HB-EGF expression level and immune gene markers in the lung cancer TGCA database. The color and sector represent Spearman’s correlation between HB-EGF and immune gene markers: **(A)** T lymphocyte markers (CTC: cytotoxic T cells, Treg: regulatory T cells, and Th: T helper cells); **(B)** B lymphocyte markers; **(C)** Mast cell markers; **(D)** Natural killer (NK) cell markers; **(E)** Neutrophil markers (Neu); **(F)** Monocyte markers; **(G)** Macrophage markers (M1 and M2 subtypes); and **(H)** Dendritic cell markers (DC, dendritic cell; cDCs, conventional dendritic cells; and pDCs, plasmacytoid dendritic cells). (LUAD, lung adenocarcinoma; LUSC, lung squamous cell carcinoma).

To determine whether poor OS in patients with LUAD is attributable to higher HB-EGF levels affecting TIIC involvement in cancer progression, we explore the correlation between the expression of HB-EGF and the marker gene sets of diverse immune cells, namely cytotoxic T cells, Tregs, T helper cells, exhausted T cells, B cells, mast cells, NK cells, neutrophils, classical monocytes, nonclassical monocytes, M1/M2 macrophages, and plasmacytoid and conventional DCs. Analysis of TCGA gene expression in lung cancer revealed that HB-EGF expression in LUAD and LUSC had no or negative correlation with the markers of T cells, B cells, mast cells, and NK cells (**Figures 2A–D**) and are mostly positively correlated with the markers of neutrophils, monocytes, and DCs (**Figures 2E–H**). Furthermore, stronger correlations were observed in LUAD than in LUSC. These results (**Figures 1**, **2**) revealed that higher HB-EGF expression might correlate with an increase in neutrophils, monocytes, and DCs in the TME, particularly in patients with LUAD with poor prognosis.

### HB-EGF expression was correlated with myeloid cells infiltration in LUAD

**Figure 3:**
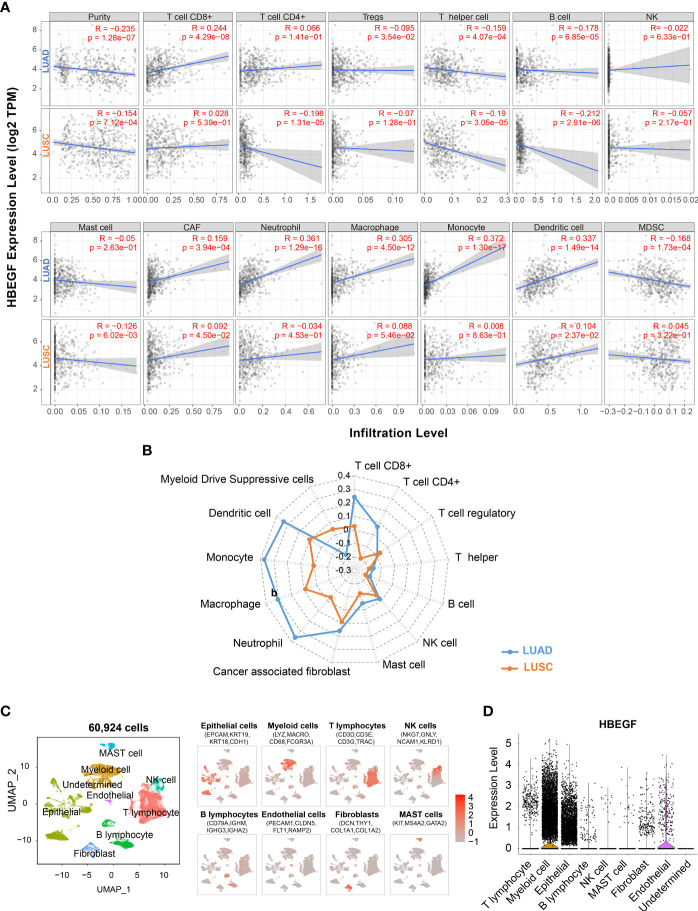
Correlation of the HB-EGF expression level with immune infiltration cells: **(A, B)** Spearman’s correlation between HB-EGF and immune infiltration cells in LUAD and LUSC using TIMER2. **(C)** UMAP plot of 60,924 cells in 15 LUAD tumor samples using single-cell RNA-seq data GSE131907, colored by eight types of major cell lineages including epithelial cells, myeloid cells, T lymphocytes, natural killer cell, B lymphocytes, endothelial cells, fibroblast, and mast cells. **(D)** The violin plot indicated HB-EGF expression across cell types in figure **(C)**.

The TIICs is an independent prognosis parameter in NSCLC (43, 44). Thus, the TIMER platform was used to determine the correlation between HB-EGF expression and TIICs in patients with NSCLC. Spearman’s rank correlation indicated that high HB-EGF expression was associated with CD8+ T cells (r = 0.244, p = 4.29e-08), macrophages (r = 0.305, p = 4.05e-12), neutrophils (r = 0.361, p = 1.29e-16), and DCs (r = 0.337, p = 1.49e-14) in LUAD (**Figures 3A, B**). Consistent with the results of the TCGA data analysis (**Figure 2**), no significant correlation was observed between HB-EGF and TIICs in LUSC.

To identify the types of cells expressing HB-EGF, we used all 15 lung tumor samples of single-cell RNA sequencing data GSE131907. We used a canonical marker set to classify 60,924 cells into eight major cell lineages (**Figure 3C** and **Supplementary Figure 3**), and the lung epithelium (e.g., alveoli and cancer cells), stroma (e.g., endothelial cells and fibroblasts), and immune cells (e.g., T, NK, B, bone marrow and mast cells) were identified as common cell types. HB-EGF was particularly highly expressed in myeloid and epithelial cells (**Figure 3D**), suggesting that these cells are the main sources of HB-EGF secretion in the lung TME. In addition, the correlation between HB-EGF and TIICs was determined using other algorithms to estimate immune cell types, namely TIMER, XCELL, MEPCOUNTER, CIBERSORT, QUANTISEQ, and EPIC (**Supplementary Table 5**). The results showed a significantly positive correlation between HB-EGF with DCs, monocytes, M2 macrophages, and neutrophils in LUAD but not in LUSC.

### HB-EGF relevant co-expressed genes were involved in chemotaxis and activation of myeloid cells

**Figure 4:**
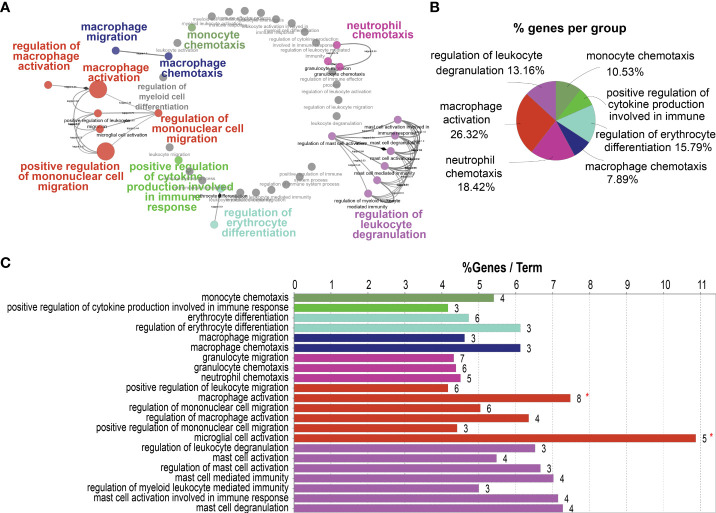
Potential pathways correlated with high HB-EGF-related genes using TCGA-LUAD: 182 genes exhibiting the highest correlation with HB-EGF were used for pathway analysis in ClueGO software. **(A)** Functionally grouped networks with terms as nodes linked based on their κ score level (≥0.3). **(B)** An overview chart with functional groups, including specific terms related to high HB-EGF expression. **(C)** The chart presents specific terms in **(B)**. The bars represent the number of genes from the analyzed cluster found to be associated with the term, and the label displayed on the bars is the percentage of identified genes compared with all genes associated with the term.

To investigate the role of HB-EGF in promoting LUAD progression, we annotated the cellular functions of HB-EGF-related genes in the TCGA-LUAD cohort by using ClueGo. We used 182 genes highly related to HB-EGF (r > 0.55, p < 0.001) for functional analysis and determined that HB-EGF may participate in various processes of the immune response, including the regulation of monocyte and macrophage migration, macrophage–monocyte chemotaxis, macrophage activation, cytokine production, neutrophil chemotaxis, and leucocyte degranulation (**Figure 4**). Most HB-EGF-related genes were associated with monocyte-macrophage chemotaxis and macrophage activation, indicating their potential role in macrophage recruitment to the TME in LUAD.

### HB-EGF promoted macrophage and lung cancer cell migration *in vitro*

**Figure 5:**
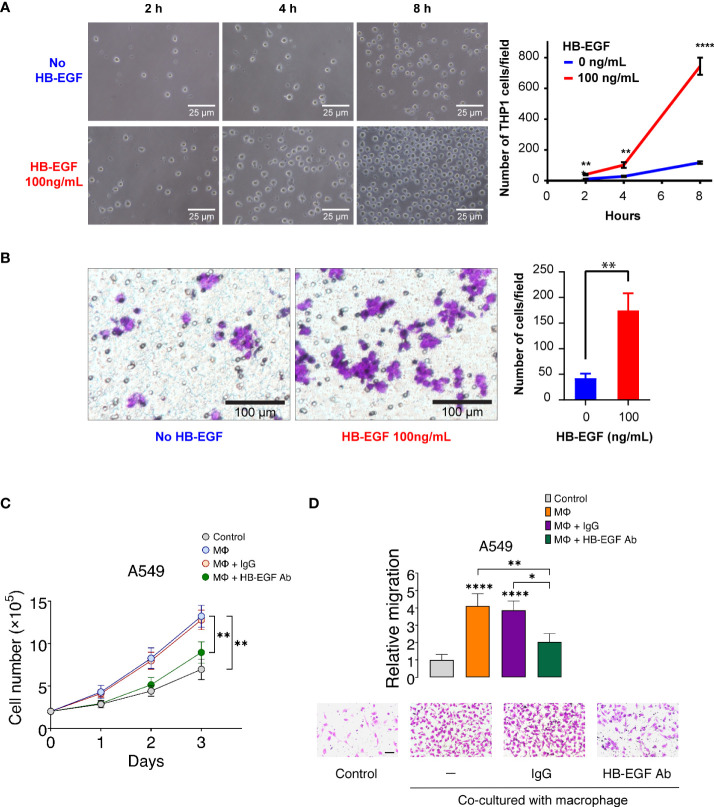
HB-EGF promotes monocyte and macrophage migration: **(A)** The short-term effect of HB-EGF mediated monocyte migration. A total of 5 × 10^5^ THP-1 cells were used in a transwell migration assay. HB-EGF was added to the lower well at a concentration of 100 ng/mL. The migration cells were observed at 2, 4, and 8 h. The right panels present representative images (magnification, 200×), the left panel shows the quantification of migrated cells, and the bar presents the mean and SD. **(B)** HB-EGF mediated macrophage migration. A total of 10^5^ THP-1 cells were differentiated into macrophages in PMA (10 ng/mL) for 48 h in the insert well of a 24-well Transwell plate prior to Transwell migration with 100 ng/mL HB-EGF for 8 h. The left panel shows representative images (magnification, 200×); the right panel presents the quantification of migrated cells, and the bar shows the standard deviation. Wilcoxon rank-sum tests were used to compare the number of migrated cells in two groups at each time point. **p < 0.01, ****p < 0.0001. **(C)** Effects of U-937-derived HB-EGF mediated the proliferation of A549 cells. U-937-derived M2 macrophages (Mϕ) were pretreated with anti-HB-EGF antibodies or control IgG for 24 h and then cocultured with A549 cells. The number of cancer cells was tracked for 1–3 days. Cell proliferation of A549 cells cocultured with Mϕ was assessed after treatment with an anti-HB-EGF neutralizing antibody. **(D)** U-937-derived HB-EGF-mediated cancer cell migration was measured. A549 cells (upper chamber) were cocultured with M2 Mϕ pretreated with anti-HB-EGF or control antibodies in transwell plates for 24 h. *p < 0.05, **p < 0.01, ****p < 0.0001.

To confirm the monocyte and macrophage chemotactic function of HB-EGF, we examined the migration ability of THP-1 monocytes and macrophages after HB-EGF treatment in the transwell migration assay. We observed that the number of migrating cells increased as early as 2 h following HB-EGF treatment (**Figure 5A**). Notably, at 8 h, we observed that cell migration increased by 6.36 times than that without HB-EGF. Consistent with our functional pathway analysis, HB-EGF significantly enhanced macrophage migration at 8 h (**Figure 5B**).

HB-EGF is secreted in U-937 cells and may be involved in macrophage-mediated cellular proliferation (45). We created a coculture system of U-937-derived macrophages and A549 cells to explore whether macrophage-derived HB-EGF affects the proliferation of lung cancer cells. The proliferation of lung cancer cells increased after coculturing with M2 macrophages (**Figure 5C**). Notably, the increase in cancer cell proliferation was significantly reduced when cocultured with macrophages treated with neutralizing HB-EGF antibodies but not reduced when cocultured with control IgG antibodies. HB-EGF secreted by TAM is closely related to primary tumor growth and promotes breast tumor migration (46). We then determined whether HB-EGF derived from macrophages-mediated lung cancer cell migration. The migration of cancer cells was significantly increased in coculture with M2 macrophages compared with the medium-only controls (**Figure 5D**). Additionally, the treatment of macrophages with anti-HB-EGF antibodies markedly diminished lung cancer cell migration compared with the control IgG. Collectively, macrophage-derived HB-EGF promoted cell proliferation and migration of lung cancer cells. Furthermore, these findings indicate that HB-EGF may increase the recruitment of TAMs and promote cancer progression.

### HB-EGF was upregulated as tumor progression and associated with the amount of M2 macrophages in a validated cohort

**Figure 6:**
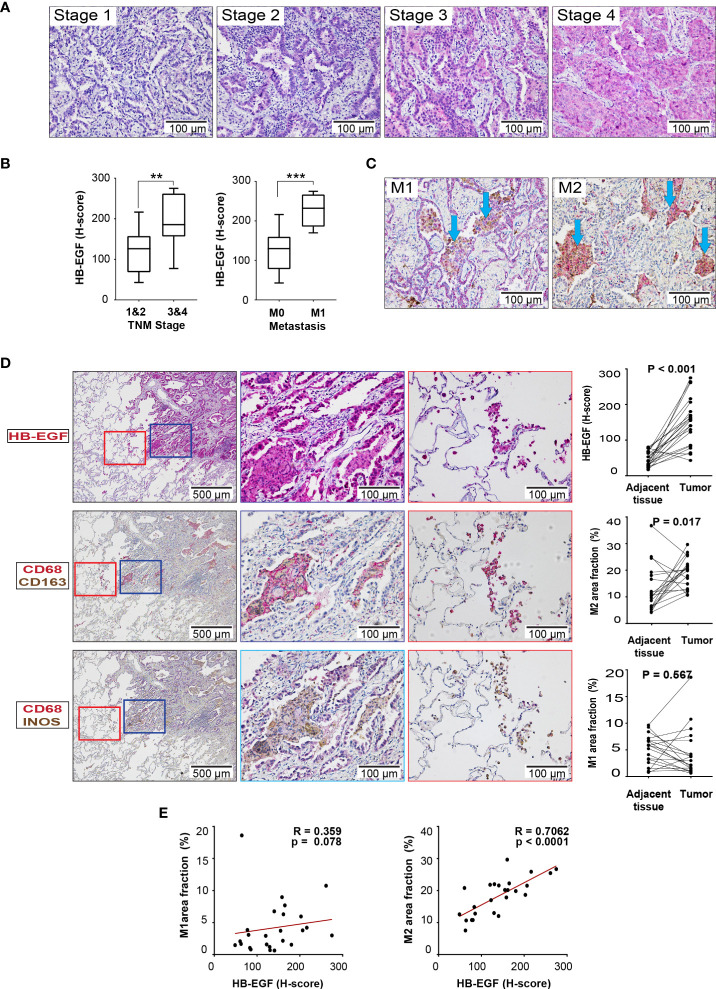
HB-EGF is highly expressed in lung cancer and positively correlated with M2 macrophages in LUAD: **(A)** Representative immunohistochemical images for HB-EGF in different stages of lung adenocarcinoma (magnification, 200×). **(B)** Semiquantitative expression of HB-EGF by stage and metastasis status. The whisker shows the standard deviation, and the Wilcoxon rank-sum test was used. **(C)** Representative immunohistochemical images for macrophage M1 and M2 double positive in lung tumor (CD68 (brown) and iNOS (red) for M1; CD68 (brown) and CD163 (red) for M2, magnification x20). **(D)** HB-EGF, M1, and M2 IHC images (magnification, 5× and 20×) and quantification for lung cancer and adjacent lung tissues. The Wilcoxon signed-rank test was used. **(E)** The dot plot shows Spearman’s correlation between HB-EGF and the fraction of M1/M2 macrophages. ** mean p < 0.01, *** mean p < 0.001.

HB-EGF protein expression was examined in the lung tissue sections derived from 30 patients with stage 1- 4 LUAD through IHC. The patients with stage 3/4 LUAD had higher tumor HB-EGF expression than did those with stage 1/2 LUAD (**Figures 6A, B**). Moreover, the patients with metastasis had higher tumor HB-EGF expression than did those without metastasis (**Figure 6B**). To evaluate the chemotactic role of HB-EGF in macrophages, M1 and M2 macrophages were stained with CD68/CD163 and CD68/iNOS, respectively (**Figure 6C**). Compared with the adjacent tissue, tumors had higher HB-EGF expression, higher M2 macrophage infiltration (**Figure 6D**), and similar M1 macrophage infiltration. Notably, HB-EGF protein expression was strongly positively correlated with M2 macrophage markers (r = 0.706, p < 0.0001) and not correlated with M1 macrophage markers (**Figure 6E**). Collectively, our findings supported a chemotactic role of tumor-expressing HB-EGF in attracting protumor M2 macrophages.

## Discussion

The cancer hallmarks include sustaining proliferative signaling, evading growth suppressors, resisting cell death, enabling replicative immortality, inducing angiogenesis, and activating invasion and metastasis (47). The ability of cancer cells to evade immune destruction is significantly associated with worse OS. By “immunoediting,” such as creating an inflammatory milieu or recruiting immunosuppressive cells to the TME, solid tumors can avoid detection and limit immune killing. Thus, immune-based therapies have the potential as cancer treatments. Since the early 2010s, the exploration of potential target-mediated cancer hallmarks through analyses of multiple transcriptional datasets has yielded powerful predictors of cancer signatures in the study of immune profiles, diagnosis, and prognosis. In our study, we used comprehensive computational methods to estimate the levels of HB-EGF expression and TIICs in lung cancer tissues by using gene expression datasets, which were validated by our independent cohort and *in vitro* experiments. The results revealed that higher HB-EGF expression in the patients with LUAD was associated with poor prognosis and an increased TIICs level, particularly in neutrophils, monocyte, macrophages, and dendritic cells. HB-EGF is mainly expressed in epithelial and myeloid cells and partly in other types of cells in the TME. Furthermore, HB-EGF was noted to promote macrophage and lung cancer migration in cell-based experiments. IHC analysis findings indicated that HB-EGF protein levels in the lung cancer tissues were significantly correlated with M2 macrophage markers. Collectively, these results illustrated that HB-EGF is markedly increased in LUAD cancer cells and may promote TIICs recruitment, particularly M2 macrophages.

In this study, six common EGFR ligands, namely TGFA, HB-EGF, AREG, EREG, EGF, and BTC, were analyzed using the gene expression datasets. Among them, HB-EGF was the most significantly expressed in NSCLC, particularly in LUAD, LUSC, and BAS. A similar result for HB-EGF expression was observed in cervical cancer (31). Our results revealed that the patients with LUAD with higher HB-EGF expression had poor OS. However, we cannot exclude the importance of other EGFR ligands in the development of LUAD. Our previous findings revealed that higher EREG expression in LUAD but not LUSC was correlated with shorter OS (48). In addition, EREG produced by TAMs causes NSCLC cell EGFR-tyrosine kinase inhibitor resistance in the TME (49). EREG and HB-EGF may mediate signaling activation through the same corresponding receptor (11). However, whether HB-EGF mediates drug resistance through TIICs, such as TAMs, remains unclear. Notably, EGFR expression is lower in LUSC than in LUAD (50), possibly cause that HB-EGF overexpression is associated with poorer prognosis in LUAD but not LUSC. Remarkably, stage-dependent TIICs in the TME may have prognostic utility for lung cancer progression (51, 52). Our analysis of the gene expression datasets revealed that HB-EGF expression in LUAD was markedly correlated with the immune marker sets of TIICs, namely monocytes, TAMs, M1/M2 macrophages, neutrophils, and DCs, but not general T cells, CD8+ T cells, Th1, Th2, Th17, or B cells. TAMs is a TIICs in the TME, and TAM-derived HB-EGF mediates cancer cell migration (46). However, M1 and M2 TAMs play tumor-suppressing and tumor/metastasis-promoting roles, respectively (53). In our study, high HB-EGF expression indicated a significant association with most M2 TAM markers and poor prognosis in patients with LUAD, indicating the role of HB-EGF in recruiting M2 TAMs in the TME. Moreover, HB-EGF expression was significantly correlated with the markers of DCs and neutrophils. DCs in the TME of lung cancer tissues derived from mice and human patients exhibited high HB-EGF levels (54). In addition, DCs in the TME not only suppress T-cell-based anticancer immune responses but also promote cancer progression, including cancer cell growth, invasion, and pro-angiogenesis (55–57). Tumor-associated neutrophils may play a tumor-promoting role in the TME in cancer progression (58). Collectively, high HB-EGF expression may play tumor-promoting roles in the TME by increasing TIICs recruitment (e.g., DCs, monocytes, macrophages, and neutrophils) in LUAD but not in LUSC.

The cross-talk between cancer and the host immune system plays a crucial role in cancer initiation and progression. HB-EGF is a chemokine for a variety of cells, such as fibroblasts (27), smooth muscle cells (28), and cancer cells (59). An analysis of single-cell RNA-seq datasets revealed a higher HB-EGF expression in myeloid and lung epithelial cancer cells. Therefore, HB-EGF secretion in cancer cells may also affect the surrounding cells by changing the TME. Consistent with the findings of IHC analysis, HB-EGF protein expression was significantly increased in lung cancer tissues compared with normal tissues. Moreover, we explored mechanisms through which HB-EGF promotes LUAD progression. ClueGo functional analysis of 182 HB-EGF highly correlated genes indicated that HB-EGF may be involved in main processes including macrophage activation, macrophage–monocyte and neutrophil chemotaxis, leukocyte degranulation, and cytokine production. Activation of the EGFR signaling in monocytes is required for cell activation and migration (14). The effect of HB-EGF expression may increase the recruitment of monocytes and macrophages and further increase cell proliferation through the MEK/ERK signaling pathway (60). Our results demonstrated the chemotactic function of HB-EGF to promote monocyte or macrophage migration following short-term HB-EGF treatment. The higher expression of HB-EGF in LUAD may be related to the accumulation of immune cells, such as TAMs, in the TME. HB-EGF released by TAMs has a strong correlation with primary tumor growth and lymph node dissemination in breast cancer (46). In addition, TAMs may increase cancer growth through the GM-CSF/HB-EGF paracrine loop (14). Similarly, our results indicated that M2 macrophage-derived HB-EGF promotes lung cancer cell proliferation and migration. Furthermore, HB-EGF also significantly enhanced macrophage migration. These findings suggest that HB-EGF may increase TAM recruitment and promote lung cancer progression. However, further studies are required to clarify the mechanisms through which the excessive accumulation of HB-EGF in the TME causes different cellular interactions.

Macrophages may play key roles in inflammation promotion and resolution, cellular damage, and tissue remodeling because M1/M2 macrophages change their functional characteristics in response to alterations in the TME (31, 57). Notably, the alteration in the immune response from the M1 to M2 phenotype may be crucial for developing new lung cancer therapeutic strategies. Macrophage activation is the pathway with the highest gene expression and is significantly associated with HB-EGF expression. In our validation cohort, we observed a significant increase in M2 macrophages in tumors compared with adjacent normal tissues. In addition, HB-EGF protein levels were significantly correlated with M2 macrophage markers. HB-EGF stimulates the repolarization of the M1 to M2 phenotype by inhibiting the STAT3 signaling pathway of LPS-mediated intestinal cell apoptosis (61). The interaction of HB-EGF with EGFR activates downstream STAT3 in the nucleus (62). Therefore, EGFR/STAT3 may be a key downstream signaling pathway for HB-EGF for promoting M2 macrophage polarization. However, whether HB-EGF promotes M2 polarization during lung cancer progression, particularly in LUAD, remains unknown.

TAMs may directly suppress cytotoxic T lymphocyte responses by upregulating immune checkpoint molecules, such as PD-L1, and inhibitory cytokine production (63). Thus, the macrophage activation status is critical in cancer progression and therapy. M2 TAMs promote tumor progression in the TME by recruiting immunosuppressive Tregs and inhibiting the remodeling of DCs in the ECM and altering the expression of numerous cytokines. Thus, HB-EGF increases M2 macrophage recruitment and may promote M2 macrophage proliferation and polarization, eventually impairing patient prognosis. Accordingly, HB-EGF suppression may be a new strategy for the treatment of certain cancers, such as LUAD.

## Conclusions

In summary, HB-EGF is highly expressed in lung cancer cells, especially LUAD, which leads to poor prognosis and is correlated with increased TIICs, including monocytes, macrophages, neutrophils, and DCs. Furthermore, the high HB-EGF expression in the TME may play a tumor-promoting role by recruiting immune cells, particularly M2 macrophages. Therefore, HB-EGF can serve as a prognostic marker and therapeutic target in patients with LUAD.

## Data availability statement

The original contributions presented in the study are included in the article/**Supplementary Material**. Further inquiries can be directed to the corresponding authors.

## Ethics statement

The study protocol was approved by the Joint Institutional Review Board of Taipei Medical University (IRB no. N202103013). The patients/participants provided their written informed consent to participate in this study.

## Author contributions

K-YL, S-MW, and NH conceptualized the study and reviewed the entire project and manuscript. NH and S-WL performed most experiments and wrote the manuscript. P-HF, S-WL, and S-MW designed the research and conducted experiments. NH, LD, and HQ performed the database collection and analysis and reviewed the manuscript. C-WL and C-SL provided expertise in statistical and figure analyses. P-HF, C-WL, and K-YC collected tumor tissue samples, conducted patient information analyses, and reviewed the manuscript. S-MW assumes responsibility for the content of the manuscript, including the data and analysis. All authors contributed to the critical revision of the manuscript for important intellectual content. All authors contributed to the article and approved the submitted version.

## Funding

This study was funded by the Ministry of Science and Technology of Taiwan (MOST: 108-2314-B-038-111-MY3, 108-2314-B-038-063-MY3, 111-2314-B-038-150-MY3, and 111-2314-B-038-152-MY3), Ministry of Education of the Republic of China (DP2-111-21121-01-T-01-01), and Taipei Medical University and Shuang Ho Hospital (110TMU-SHH-19).

## Conflict of interest

The authors declare that the research was conducted in the absence of any commercial or financial relationships that could be construed as a potential conflict of interest.

## Publisher’s note

All claims expressed in this article are solely those of the authors and do not necessarily represent those of their affiliated organizations, or those of the publisher, the editors and the reviewers. Any product that may be evaluated in this article, or claim that may be made by its manufacturer, is not guaranteed or endorsed by the publisher.

## Supplementary material

Supplementary Figure 1 Overexpression of HB-EGF in lung cancer. **(A)** The mRNA expression levels of the EGFR ligands, including heparin-binding EGF-like growth factor (HB-EGF), epidermal growth factor (EGF), transforming growth factor-a (TGFA), betacellulin (BTC), amphiregulin (AREG), and epiregulin (EREG) in lung cancer were analyzed by using GSE3141 and GSE50081 dataset. The levels of HB-EGF were compared with other ligands by using Wilcoxon rank-sim test, ****P*<0.001; **(B–D)** The dot-plot showed Spearman’s correlation between EGFR and its ligands in GSE30219, GSE3141, and GSE50081 datasets.

Supplementary Figure 2 Kaplan–Meier plot analysis of Progression free survival (PFS) according to the HB-EGF mRNA expression level in patients with LUAD **(A)** and LUSC **(B)** using combine 4 GEO datasets including GSE29013, GSE31210, GSE50081 and GSE8894. We calculated p values by using the log-rank test.

Supplementary Figure 3 The dot-plot of selected marker for 8 cell types using single-cell RNA sequencing data GSE131907. Epithelial cells markers: EPICAM, KTR18/19, CDH1; Fibroblast cells markers: DCN, THY1, COL1A1/2; Endothelial markers: PECAM1, CLDN5, FLT1, RAMP2, T cell’s markers: CD3D/E/G, TRAC; Natural killer cells markers: NKG7, GNLY, NCAM1, KLRD1; B cells markers: CD79A, IGHM, IGHG3, IGHA2; Myeloid cells markers: LYZ, MARCO, CD68, FCGR3A; Mast cells markers: KIT, MS4A2, GATA2.
