# Corrigendum: Heparin binding epidermal growth factor–like growth factor is a prognostic marker correlated with levels of macrophages infiltrated in lung adenocarcinoma

**DOI:** 10.3389/fonc.2022.1106553

**Published:** 2022-12-13

**Authors:** Nguyen Van Hiep, Wei-Lun Sun, Po-Hao Feng, Cheng-Wei Lin, Kuan-Yuan Chen, Ching-Shan Luo, Le Ngoc Dung, Hoang Van Quyet, Sheng-Ming Wu, Kang-Yun Lee

**Affiliations:** ^1^ International Ph.D. Program in Medicine, College of Medicine, Taipei Medical University, Taipei, Taiwan; ^2^ Oncology Center, Bai Chay Hospital, Quang Ninh, Ha Long, Vietnam; ^3^ Department of Thoracic and Neurological Surgery, Bai Chay Hospital, Quang Ninh, Ha Long, Vietnam; ^4^ Division of Pulmonary Medicine, Department of Internal Medicine, Shuang Ho Hospital, Taipei Medical University, New Taipei City, Taiwan; ^5^ Division of Pulmonary Medicine, Department of Internal Medicine, School of Medicine, College of Medicine, Taipei Medical University, Taipei, Taiwan; ^6^ TMU Research Center for Thoracic Medicine, Taipei Medical University, Taipei, Taiwan; ^7^ Department of Biochemistry and Molecular Cell Biology, School of Medicine, College of Medicine, Taipei Medical University, Taipei, Taiwan; ^8^ Graduate Institute of Clinical Medicine, College of Medicine, Taipei Medical University, Taipei, Taiwan

**Keywords:** heparin-binding EGF-like growth factor (HBEGF), bioinformatics, biomarker, immune infiltration, macrophage chemotaxis, non-small cell lung cancer

In the published article, there was an error in affiliation 1. Instead of “IInternational Ph.D. Program in Medicine, College of Medicine, Taipei Medical University, Taipei, Taiwan”, it should be “International Ph.D. Program in Medicine, College of Medicine, Taipei Medical University, Taipei, Taiwan”.

In the published article, there was an error in the Ethics statement. The IRB number was incorrectly displayed as “IRB no. N20210313”. The correct IRB number is “IRB no. N202103013”. The correct Ethics statement appears below.

## Ethics statement

The study protocol was approved by the Joint Institutional Review Board of Taipei Medical University (IRB no. N202103013). The patients/participants provided their written informed consent to participate in this study.

The authors apologize for these errors and state that this does not change the scientific conclusions of the article in any way. The original article has been updated.

