# Supplementary figures and images for: Heparin binding epidermal growth factor–like growth factor is a prognostic marker correlated with levels of macrophages infiltrated in lung adenocarcinoma
Source: Front Oncol. 2022 Nov 10;12:963896. doi: 10.3389/fonc.2022.963896 (PMC9686304; doi:10.3389/fonc.2022.963896)

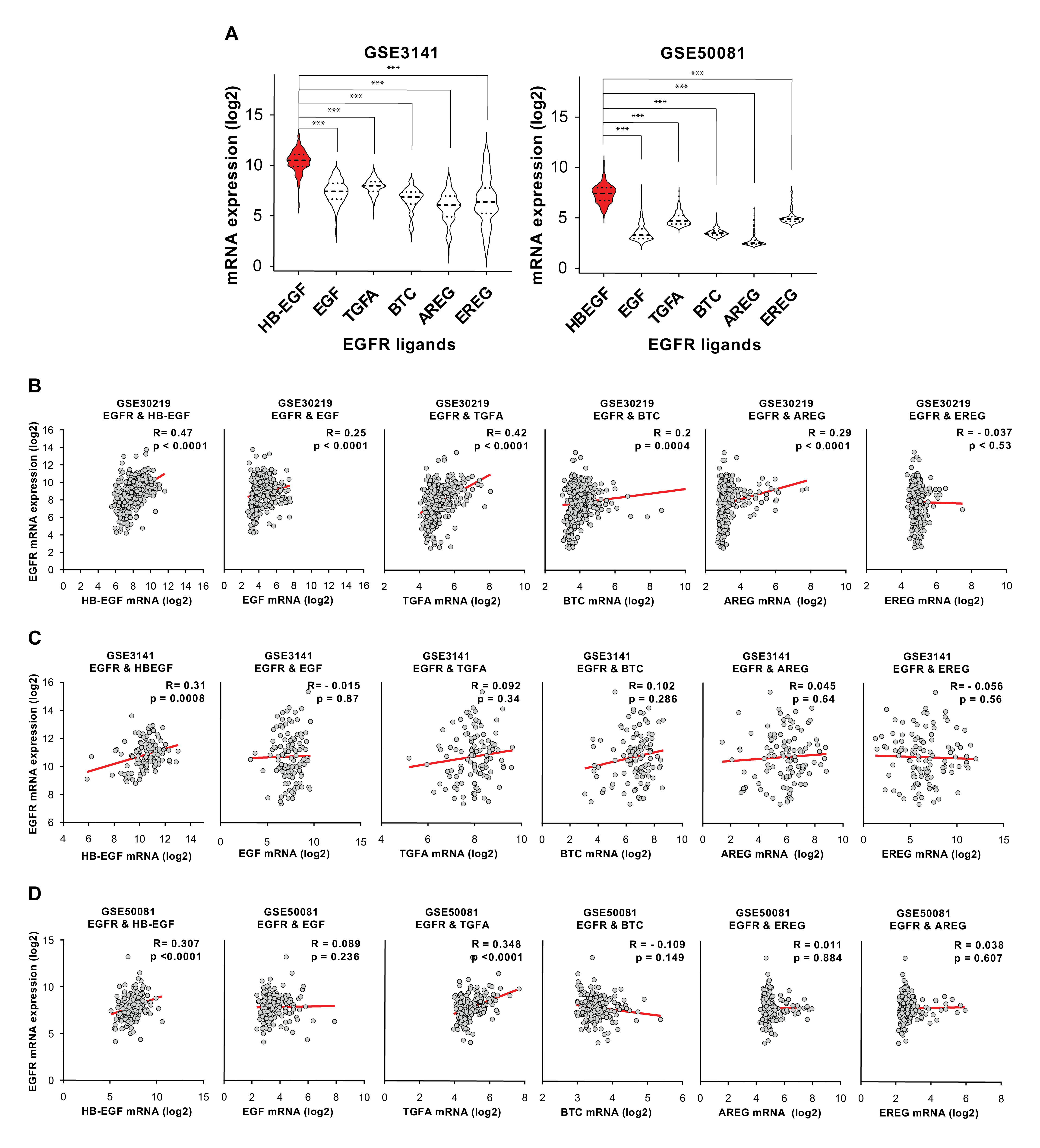

Supplement: Supplementary Figure 1 — Overexpression of HB-EGF in lung cancer. (A) The mRNA expression levels of the EGFR ligands, including heparin-binding EGF-like growth factor (HB-EGF), epidermal growth factor (EGF), transforming growth factor-a (TGFA), betacellulin (BTC), amphiregulin (AREG), and epiregulin (EREG) in lung cancer were analyzed by using GSE3141 and GSE50081 dataset. The levels of HB-EGF were compared with other ligands by using Wilcoxon rank-sim test, ***P<0.001; (B–D) The dot-plot showed Spearman’s correlation between EGFR and its ligands in GSE30219, GSE3141, and GSE50081 datasets. [file Image_1.tif]

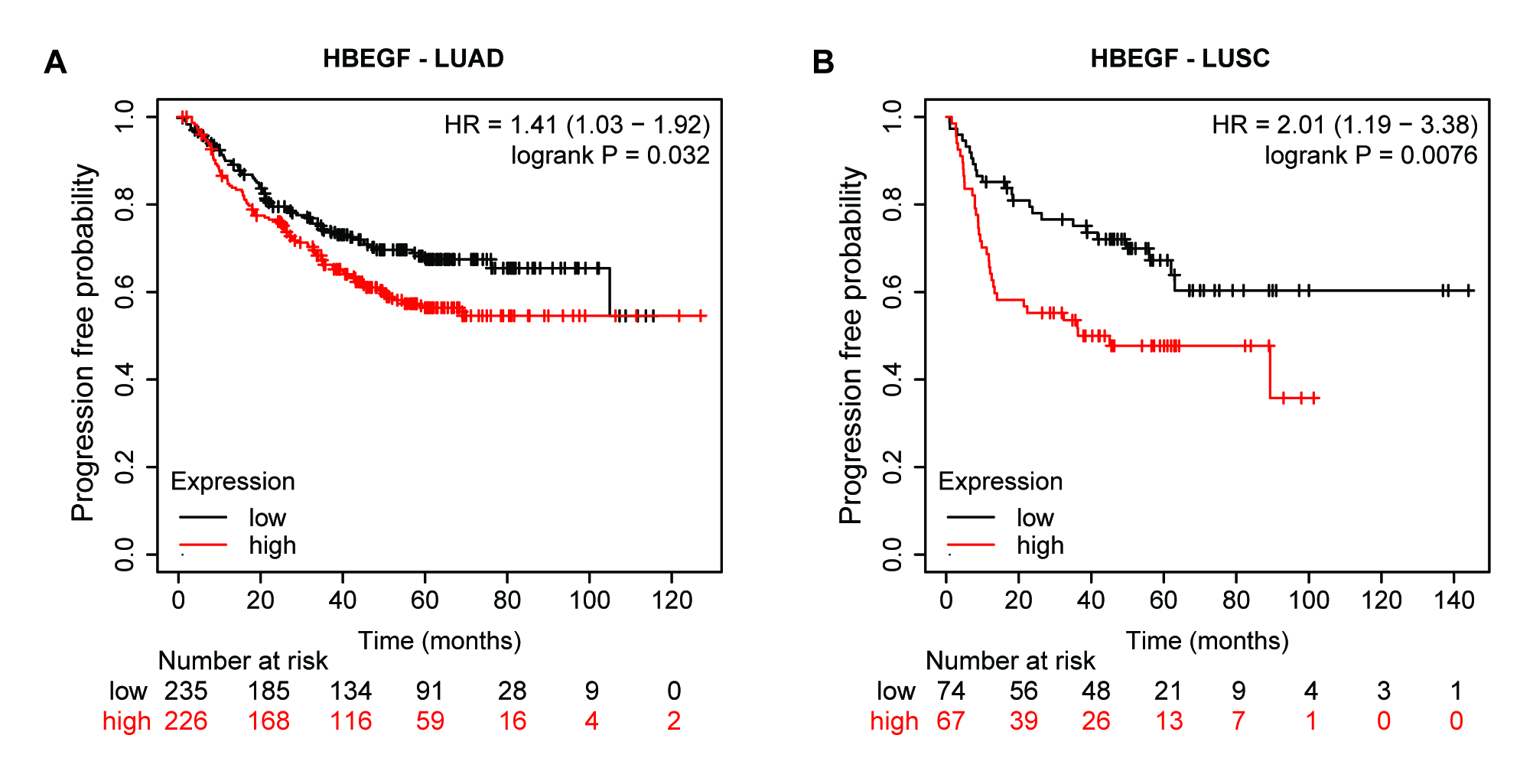

Supplement: Supplementary Figure 2 — Kaplan–Meier plot analysis of Progression free survival (PFS) according to the HB-EGF mRNA expression level in patients with LUAD (A) and LUSC (B) using combine 4 GEO datasets including GSE29013, GSE31210, GSE50081 and GSE8894. We calculated p values by using the log-rank test. [file Image_2.tif]

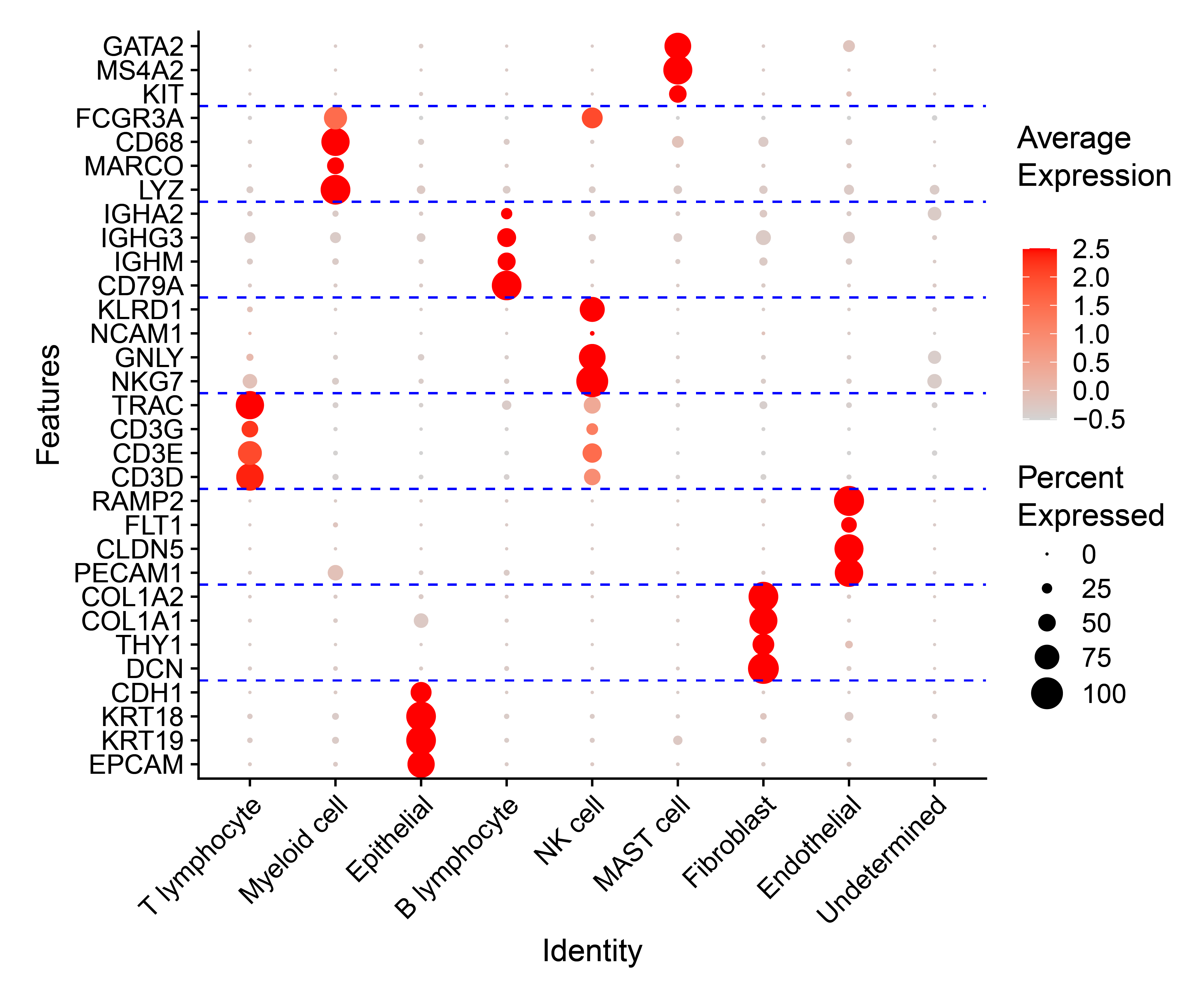

Supplement: Supplementary Figure 3 — The dot-plot of selected marker for 8 cell types using single-cell RNA sequencing data GSE131907. Epithelial cells markers: EPICAM, KTR18/19, CDH1; Fibroblast cells markers: DCN, THY1, COL1A1/2; Endothelial markers: PECAM1, CLDN5, FLT1, RAMP2, T cell’s markers: CD3D/E/G, TRAC; Natural killer cells markers: NKG7, GNLY, NCAM1, KLRD1; B cells markers: CD79A, IGHM, IGHG3, IGHA2; Myeloid cells markers: LYZ, MARCO, CD68, FCGR3A; Mast cells markers: KIT, MS4A2, GATA2. [file Image_3.tif]
